# Supplementary material for: Cholinergic Receptor Nicotinic Beta 2 Subunit Promotes the Peritoneal Disseminating Metastasis of Colorectal Cancer
Source: Cancers (Basel). 2025 Jul 28;17(15):2485. doi: 10.3390/cancers17152485 (PMC12346510; doi:10.3390/cancers17152485)

Supplementary Table S1. Specific primers for polymerase chain reaction assays for *CHRNB2* and *GAPDH*

	Experiment	Type	Sequence (5´ - 3´)	Product size	Annealing temperature
CHRNB2	qRT-PCR	forward	AGCGAGGACGATGACCAG	105 bp	60℃
		reverse	GGTGCCAAAGACACAGACAA		
GAPDH	qRT-PCR	forward	GAAGGTGAAGGTCGGAGTC	226 bp	60℃
		probe	CAAGCTTCCCGTTCTCAGCC		
		reverse	GAAGATGGTGATGGGATTTC		

CHRNB2, cholinergic receptor nicotinic beta 2 subunit

GAPDH, glyceraldehyde-3-phosphate dehydrogenase

RT-PCR, reverse transcription polymerase chain reaction; bp, base pair; si-RNA, small interfering RNA.

Supplementary Table S2. Association between expression level of CHRNB2 mRNA and clinicopathological parameters of 301 colorectal cancer patients.

**Clinicopathological parameters**	**Low CHRNB2 (n = 226)**	**High CHRNB2 (n = 75)**	***P***
Age			
< 65 years	72	31	0.137
≥ 65 years	154	44	
Gender			
Male	124	35	0.217
Female	102	40	
Tumor location			
Right	75	18	0.129
Left	151	57	
Tumor size (mm)			
< 40	109	36	0.972
≥ 40	117	39	
Carcinoembryonic antigen (ng/ml)			
≤ 5	154	45	0.200
> 5	72	30	
Carbohydrate antigen 19-9 (IU/ml)			
≤ 37	172	59	0.647
>37	54	16	
Tumor depth			
pT1, 2, 3	192	55	0.093
pT4	34	19	
Lymphatic involvement			
Absent	66	14	0.073
Present	160	61	
Vascular invasion			
Absent	88	26	0.490
Present	137	49	

Pathological stage			
II	117	38	0.834
III	110	37	

CHRNB2, cholinergic receptor nicotinic beta 2 subunit.

Supplementary Figure S1. Kaplan-Meier plotter analysis for OS and DFS in 1002 CRC patients. (a) Disease-free survival rate (b) Overall survival rate.

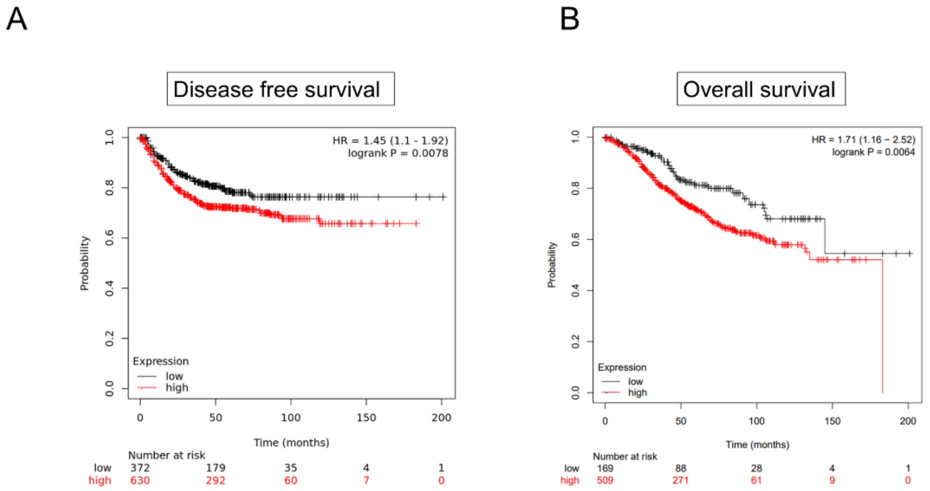

Supplement: Supplementary file 1 [file cancers-17-02485-s001.zip › cancers-3749996-supplementary.pdf]
